# 2149. Risk of dental abnormalities after tetracycline exposure in children: A retrospective, population-based study in Korea, 2003-2015

**DOI:** 10.1093/ofid/ofac492.1769

**Published:** 2022-12-15

**Authors:** SeoJung Kim, Eun Hwa Kim, Myeongjee Lee, Je Hee Shin, Sung Min Lim, In Kyung Jung, Jong Gyun Ahn, Ji-Man Kang, Chung-Min Kang, Jee Yeon Baek, Ji Young Lee

**Affiliations:** Severance Children’s Hospital, Yonsei University College of Medicine, Seoul, Seoul-t'ukpyolsi, Republic of Korea; Yonsei University College of Medicine, Seoul, Seoul-t'ukpyolsi, Republic of Korea; Yonsei University College of Medicine, Seoul, Seoul-t'ukpyolsi, Republic of Korea; Severance Children’s Hospital, Yonsei University College of Medicine, Seoul, Seoul-t'ukpyolsi, Republic of Korea; Severance Children’s Hospital, Yonsei University College of Medicine, Seoul, Seoul-t'ukpyolsi, Republic of Korea; Yonsei University College of Medicine, Seoul, Seoul-t'ukpyolsi, Republic of Korea; Severance Children’s Hospital, Yonsei University College of Medicine, Seoul, Seoul-t'ukpyolsi, Republic of Korea; Severance Children’s Hospital, Yonsei University College of Medicine, Seoul, Seoul-t'ukpyolsi, Republic of Korea; Yonsei University College of Dentistry, Seoul, Seoul-t'ukpyolsi, Republic of Korea; Yonsei University College of Medicine, Seoul, Seoul-t'ukpyolsi, Republic of Korea; Yonsei University College of Medicine, Seoul, Seoul-t'ukpyolsi, Republic of Korea

## Abstract

**Background:**

Despite its clinical usefulness, the use of tetracycline antibiotics (TCs) in children has historically been limited because of the risk of permanent tooth discoloration and enamel deformity, although age restrictions have varied across countries (e.g., contraindicated under 8 years of age in Taiwan, USA, and Canada, and under 12 years of age in Korea and UK). We explored whether the incidence of dental abnormalities in Korean children with a history of TCs use differed by age. Furthermore, the relative risk compared to the TCs non-exposed group was calculated.

**Methods:**

From 2002 to 2015, 1 million standard sample subjects data was provided from National Health Insurance Service in Korea. Children younger than 18 years were included and divided into groups A (0-7 years), B (8-12 years), and C (13-17 years). Subjects in 2002 were excluded for wash-out, as were those diagnosed with dental abnormalities within 6 months after TCs prescription. For comparison, 1:4 matching between the TCs exposure and non-exposure groups was performed according to age and gender.

**Results:**

Among 14,831 individuals included as study subjects (487 (3%) in group A; 1,695 (11%) in group B; and 12,649 (85%) in group C), 201 (1.4%) were diagnosed with dental abnormalities. The 5-year cumulative incidence of dental abnormalities after exposure to TCs in the group A was 4.7%, which was significantly higher than that of the control group C (0.9%; p< 0.0001), but that of group B was 1.3% and not significantly different from that of group C. In the risk analysis of dental abnormalities according to exposure of TCs, there was no significant difference (adjusted RR=1.03, 95%CI=0.71 to 1.50 in group A; adjusted RR=1.13, 95%CI=0.76 to 1.69 in group B; adjusted RR=1.18, 95%CI=0.97 to 1.43 in group C).

**Fig 1.**
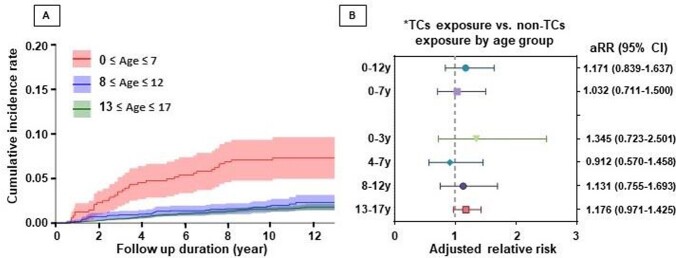
Cumulative incidence rate and relative risk analysis.

**Conclusion:**

Korean children aged 0-7 years who were exposed to TCs had a higher cumulative incidence and relative risk of dental deformities compared to the 13-17-year-old group but not the 8-12-year-old group. No significant increase in dental abnormalities was observed with TCs exposure among pediatric age groups.

**Disclosures:**

**All Authors**: No reported disclosures.

**Fig 1.** Cumulative incidence rate and relative risk analysis.

A. Cumulative incidence rate of dental abnormalities after TCs exposure by age group (p = <.00001). B. Relative risk analysis by TCs exposure and age group.

Abbreviation; TCs: tetracycline and its derivatives.

